# RURAL–URBAN DIFFERENCES IN INFORMAL CAREGIVER RESOURCES, CAREGIVING INTENSITY, AND HEALTH-RELATED QUALITY OF LIFE

**DOI:** 10.1093/geroni/igad104.1388

**Published:** 2023-12-21

**Authors:** Steven Cohen, Neelam Ahmed, Mary Greaney

**Affiliations:** University of Rhode Island, Kingston, Rhode Island, United States; Brown University School of Public Health, Providence, Rhode Island, United States; University of Rhode Island, Kingston, Rhode Island, United States

## Abstract

Rural-urban health disparities are pervasive among older adults and their caregivers. Rural areas in the US have a disproportionately high population of older adults, have reduced access to services, and are more reliant on family and friends for care. Research examining rural-urban disparities for informal caregivers is limited. There is a critical need to understand how caregiving intensity, access to resources, and caregiver health-related quality of life (HRQoL) differ by rural-urban status. Study objectives were to explore rural-urban differences in caregiving resources, intensity, and HRQoL. Data were abstracted from Waves 1 and 5 of the National Study of Caregiving. Rural-urban status was based on Rural Urban Continuum Codes. Generalized linear models, accounting for complex sampling, were used to explore associations between rural-urban status and caregiver resource utilization, caregiving intensity, and HRQoL. Compared to urban caregivers, rural caregivers were significantly more likely to be female (OR 1.19, 95%CI 1.12-1.26), older (beta=1.13 years, 95%CI 0.70-1.55), have obesity (OR 1.25, 95%CI 1.17-1.32), report poor/fair health (OR 1.08, 95%CI 1.01-1.16), and higher numbers of comorbidities (beta=0.18, 95%CI 0.13-0.22). Compared to urban caregivers, rural caregivers were significantly less likely to attend support groups (OR 0.55, 95% CI 0.46-0.65), but more likely to have caregiver training (OR 1.46, 95%CI 1.33-1.62). They assisted with fewer instrumental activities of daily living (beta=0.54, 95%CI 0.44-0.65), although there was not a significant difference for activities of daily living. Findings can facilitate the translation and adaption of policies, programs, and interventions to support and address the unique needs of rural caregivers.

